# Social network diversity and COVID-19 infection and severity risk: a longitudinal population study

**DOI:** 10.3389/fpubh.2025.1730268

**Published:** 2026-01-13

**Authors:** Takahiro Suzuki, Takeo Fujiwara, Takahiro Tabuchi

**Affiliations:** 1 Department of Public Health, Institute of Science Tokyo, Tokyo, Japan; 2 Department of Cardiovascular Medicine, St. Luke’s International Hospital, Tokyo, Japan; 3 Center for Wellbeing Research Advancement, Institute of Future Science, Institute of Science Tokyo, Tokyo, Japan; 4 Division of Epidemiology, School of Public Health, Tohoku University Graduate School of Medicine, Sendai, Japan

**Keywords:** social network diversity, coronavirus disease 2019, social determinants of health, longitudinal cohort study, infection

## Abstract

**Background:**

Clinical evidence on how social network diversity (SND) influences the risk of infection and disease severity during the coronavirus disease 2019 (COVID-19) pandemic remains limited. We aim to investigate the associations between SND and the risk of COVID-19 infection and disease severity using a large-scale longitudinal cohort study.

**Methods:**

We analyzed data from participants in a longitudinal study, the Japan COVID-19 and Society Internet Survey (JACSIS) between 2020 and 2023. The SND score was calculated as the sum of seven distinct types of social networks. COVID-19 infection was assessed as ever infection, and severity was defined as oxygen-requiring admission, using a self-reported questionnaire. Poisson regression with robust standard errors estimated risk ratios (RRs) and 95% confidence intervals (CIs), adjusting for sociodemographic and clinical characteristics.

**Results:**

Of 13,713 participants (mean age 53.2 ± 15.7 years, 46.4% women), 3,251 (23.7%) developed COVID-19, and among infected individuals, 277 (8.5%) required oxygen therapy. Higher SND scores were associated with COVID-19 infection with linear trend (SND score 7 vs. 0: adjusted RR 2.49; 95% CI 2.11–2.95). In contrast, the association between SND score and disease severity followed a U-shaped pattern, with 4–5 SND showing the lowest risk of oxygen-requiring admission (adjusted RR 0.15; 95% CI 0.11–0.30) compared to those with 0 SND.

**Conclusion:**

While higher SND was associated with increased COVID-19 infection risk, moderate social network diversity appeared protective against severe disease outcomes. These findings suggest a complex trade-off between exposure risk and potential health benefits of social networks during infectious disease outbreaks.

## Introduction

The coronavirus disease 2019 (COVID-19) pandemic has underscored the unprecedented threat that emerging infectious diseases can pose to global healthcare systems (1). This crisis not only demonstrated the urgency of implementing effective public health measures but also emphasized the need to understand better the factors that shape both societal and individual vulnerability to infectious diseases (2). One noteworthy factor is the association between social connections and health outcomes, as previous epidemiological research suggests that individuals with stronger and more diverse social networks tend to demonstrate superior health outcomes (3, 4).

Existing research on the association between social network diversity (SND) and infectious disease risk has shown inconsistent and sometimes contradictory findings. Some studies showed that diverse social networks improve health outcomes, while others have reported positive associations between SND and adverse outcomes, including increased infection risk. In respiratory infections, Cohen et al.’s study showed that individuals with extensive social networks were less likely to develop symptoms after experimental rhinovirus exposure (5). Conversely, other studies have documented that greater SND is associated with an elevated risk of infection, presumably through increased exposure to pathogens from diverse social contacts (6).

These findings highlight a fundamental tension in understanding how social connections influence infectious disease outcomes. While increased social interaction inevitably enhances exposure opportunities, robust social networks may simultaneously attenuate disease severity through stress-buffering effects and enhanced immune function (7, 8). The presence of these opposing effects underscores the importance of systematically evaluating how social network diversity influences both infection and disease severity risk, particularly for novel pathogens characterized by high transmissibility and severe disease potential, such as COVID-19. Given the conflicting evidence from prior research and the unique characteristics of the COVID-19 pandemic, there is a critical need to rigorously examine whether social network diversity differentially affects infection risk versus disease severity, and to elucidate the potential mechanisms underlying these associations. Therefore, we aimed to investigate the associations between social network diversity and the risk of COVID-19 infection and disease severity using a large-scale contemporary longitudinal cohort from the early stages of the COVID-19 pandemic.

## Methods

### Study setting and population

We analyzed data from the Japan COVID-19 and Society Internet Survey (JACSIS), a comprehensive web-based longitudinal survey designed to approximate a nationally representative distribution of adults in Japan. Specifically, this investigation included two iterations of the JACSIS survey: 2020 and 2023. In the 2020 wave, 224,389 registered panellists aged 15–79 years were initially contacted through random sampling, stratified by sex, age, and prefecture, thereby encompassing all 47 prefectures. Between August and September 30, 2020, a total of 28,000 individuals completed the questionnaire. Subsequently, in the 2023 wave, 59,219 individuals—some of whom had participated in 2020—were invited, and responses were collected from September to November, 2023, resulting in 33,000 participants. Japan experienced eight COVID-19 waves from 2020–2023: initial outbreak (March 2020), summer surge (July–August 2020), winter peak (November 2020–February 2021), Alpha variant (March–June 2021), Delta variant (August 2021), Omicron variant (early 2022) with highest cases but lower hospitalization rates, followed by two additional waves in summer and winter 2022 (9). Japan implemented a series of “states of emergency” rather than mandatory lockdowns, focusing on cluster-based contact tracing and voluntary behavioral changes (10). Japan’s vaccination campaign began in February 2021, reaching over 80% of the population with two doses by late 2022 (11, 12).

As a token of appreciation, each participant received electronic reward points (“E-points”) upon completion, and the option to withdraw at any point was clearly communicated to them. All respondents provided informed consent via an online portal before participating. The JACSIS study protocol received ethical clearance from the Research Ethics Committee of the Osaka International Cancer Institute (approval number 20084). We followed the Strengthening the Reporting of Observational Studies in Epidemiology (STROBE) reporting guideline.

### Outcome measures and definition of social network score

The primary outcome was the incidence of COVID-19 infection. Additionally, for participants who developed COVID-19 infection, the severity was defined the necessity of hospital admission for COVID-19 infection requiring oxygen. We defined severity of COVID-19 infection as admission requiring oxygen because it represents an established clinical indicator of disease severity that has been validated in previous studies (13–15). The incidence of COVID-19 infection was ascertained through self-reported questionnaires that assessed both infection history and the requirement for oxygen during hospitalization. The exact questions were: “Have you experienced an event where you were diagnosed with COVID-19?” and “Have you experienced an event where you were infected with COVID-19 and received oxygen therapy requiring hospitalization?”

The exposure of interest was the Social Network Diversity. We extracted nine items from the JACSIS questionnaire, which were subsequently grouped into seven categories, as previously described in detail elsewhere (5). Specifically, these variables were defined as direct interpersonal contact, including: (1) cohabiting or sharing finances with another person; (2) working in an office with at least one other individual; (3) interacting with neighbors; (4) face-to-face meetings with non-cohabiting family or relatives; (5) face-to-face meetings with friends or acquaintances; (6) participation in volunteer groups; (7) in-person participation in sports-related groups or circles; (8) in-person participation in hobby-, learning-, or culture-oriented groups; and (9) attendance at gatherings organized by municipalities or social welfare councils (e.g., salons). Responses for each item were offered on a seven-point frequency scale includes: almost every day (6–7 times a week), 4–5 times a week, 2–3 times a week, once a week, 2–3 times a month, once a month, and rarely.

To quantify SND, we assigned one point to each item if participants reported engaging in that activity at least once a month and zero points otherwise, following previous research that emphasizes the diversity (i.e., the number of different types) of social connections. Items 1 through 5 and item 9 were scored individually. In contrast, items 6, 7, and 8 (volunteer, sports-related, and hobby/learning/cultural groups) were combined into a single category, given their shared nature as organized group activities; participants were assigned one point if they engaged in any of these at least once per month. The total SND score therefore ranged from 0 to 7, with higher scores indicating greater diversity in social network participation (5).

### Covariates

In addition to basic participant characteristics, including age, sex, and body mass index (BMI), the questionnaire included comorbid conditions—hypertension, diabetes mellitus, asthma, and chronic obstructive pulmonary disease (COPD)— as binary variables, following previous studies (16, 17). Socioeconomic and lifestyle factors, which need to be adjusted because they have been consistently associated with variations in health outcomes and may confound the relationship between social determinants and disease severity (18), were also collected: income was classified into low, intermediate, or high categories, while smoking status (current or non-current), and alcohol consumption (none/light vs. moderate/heavy) were based on self-report. Housing status was categorized as owning one’s home, renting, or others. Educational level was grouped as “high school or lower” or otherwise, and employment status was divided into full-time, part-time, and unemployed/student. Living arrangement was classified as “living alone” or “living with others,” and marital status as married, unmarried, or others. These covariates were selected *a priori* to control for sociodemographic disparities, health behaviors, and chronic disease histories that may confound the relationship between the SND score and clinical outcomes.

### Statistical analysis

Continuous variables are presented as the mean ± standard deviation (SD), and categorical variables are presented as counts and proportions. Baseline participant characteristics were compared between those with and without Social networks using the Student’s t-test for continuous variables and the Chi-square test for categorical variables. To evaluate potential selection bias, we compared the baseline characteristics of these eligible participants with those excluded or lost to follow-up. Given the large sample size, baseline characteristics were analyzed using standardized differences, where an absolute difference of more than 10 percent was considered important.

First, to provide an unadjusted descriptive view of event rates, we constructed bar charts illustrating the proportion of each outcome (COVID-19 incidence or oxygen-requiring admission) at each level of the social network score. Next, to flexibly capture potential nonlinear relationships between the SND score and each outcome, we visualized the associations using restricted cubic splines analysis. The optimal number of knots for the spline functions was determined by minimizing the Akaike Information Criterion (AIC), balancing model fit and complexity. Three models were constructed: Model 1 (unadjusted), Model 2 (adjusted for age, age-squared and sex), and Model 3 (adjusted for age, age-squared, sex, BMI, BMI-squared, hypertension, diabetes, asthma, COPD, income, smoking status, alcohol use, housing, and education level). To quantify these relationships, we employed Poisson regression models with robust standard errors (using sandwich variance estimators) to estimate risk ratios (RRs) and their 95% confidence intervals, with a social network score of 0 serving as the reference point. Additionally, to statistically test for non-linearity, we included quadratic terms for the SND score in our regression models. The significance of the quadratic term coefficient was assessed using robust standard errors to confirm the presence of non-linear relationships between SND and outcomes. There were no missing values in Models 1 and 2. In Model 3, 2,785 (20.3%) participants had missing values in socioeconomic variables (income and education), and we performed a complete case analysis. Details on the directed acyclic graph (DAG) are provided to illustrate the rationale for variable selection (Supplementary Figure 1). In addition, sensitivity analyses stratified by age and sex were conducted to evaluate possible interactions. Statistical significance was set at *α* = 0.05, and all analyses were conducted using R version 4.2.3 (R Foundation for Statistical Computing, Vienna, Austria).

## Results

**Figure 1:**
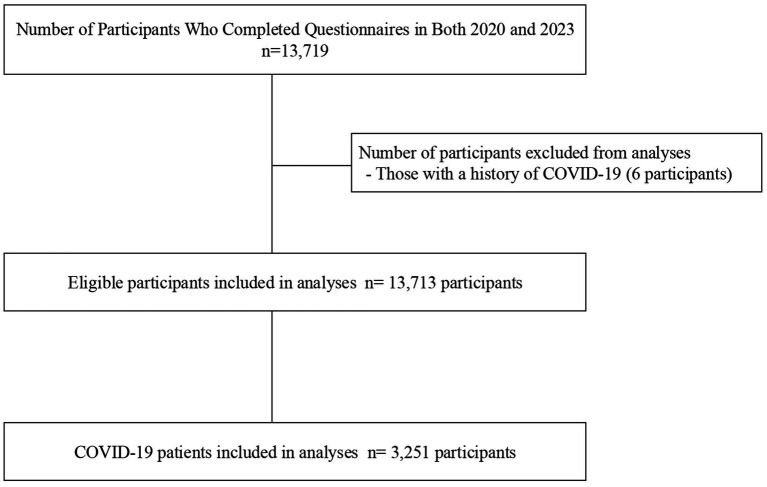
Flow chart of the study. Study population and eligibility criteria are shown. COVID-19, Coronavirus disease 2019.

Consecutive 13,719 participants who underwent questionnaires from 2020 to 2023 wave were included in the study. We excluded participants with a history of COVID-19 (n = 6), leading to a final eligible cohort of 13,713 participants (Figure 1). As shown in Supplementary Table 1, the eligible participants were substantially older (mean age 53.2 vs. 44.4 years), had a lower proportion of women (46.4% vs. 53.8%), and were more likely to own their homes compared to the excluded group. Among the eligible participants, the mean age was 53.2 ± 15.7 years, and 6,357 (46.4%) were women. There were no missing data for the primary outcome and SND score. The median SND score was 3 [Q1-Q3: 2–5], with 225 participants (1.6%) reporting an SND score of 0, and 487 participants (3.6%) reporting the highest SND score of 7 (Supplementary Figure 2). Table 1 presents baseline characteristics and procedural information stratified by SND score, including the number of missing values for each variable. Participants with higher SND scores tended to be younger, had higher income levels, and were more likely to own their homes compared to those with lower scores (Table 1). In this cohort, 27.7% of participants had hypertension, 8.9% had diabetes, 1.8% had chronic obstructive pulmonary disease (COPD), 11.6% had asthma, and 40.3% were smokers.

**Table 1:** Baseline characteristics of study participants, stratified by social network diversity score.

Characteristics	0	1	2	3	4	5	6	7	SMD (Percentage Points)
Number of the participants	225	1,491	2,900	2,763	2,779	2055	1,013	487	
Age (years), mean (SD)	56.2 (14.6)	52.2 (15.6)	50.6 (15.5)	52.7 (15.5)	54.4 (15.7)	57.0 (14.7)	55.1 (16.2)	45.0 (15.4)	28.4
Female gender, No. (%)	71 (31.6)	671 (45.0)	1,214 (41.9)	1,360 (49.2)	1,470 (52.9)	1,041 (50.7)	391 (38.6)	139 (28.5)	22.2
Body Mass Index, mean (SD) kg/m^2^	22.9 (4.2)	22.6 (5.3)	22.5 (4.0)	22.6 (8.7)	22.6 (8.1)	22.6 (7.8)	23.2 (4.2)	22.5 (3.8)	3.2
Previous medical history									
Hypertension, No. (%)	62 (27.6)	370 (24.8)	712 (24.6)	766 (27.7)	779 (28.0)	638 (31.0)	325 (32.1)	144 (29.6)	7.2
Diabetes, No. (%)	31 (13.8)	147 (9.9)	236 (8.1)	219 (7.9)	229 (8.2)	184 (9.0)	102 (10.1)	69 (14.2)	9.0
COPD, No. (%)	9 (4.0)	28 (1.9)	51 (1.8)	33 (1.2)	28 (1.0)	27 (1.3)	32 (3.2)	36 (7.4)	12.9
Asthma, No. (%)	30 (13.3)	145 (9.7)	328 (11.3)	292 (10.6)	334 (12.0)	247 (12.0)	131 (12.9)	80 (16.4)	7.1
Income, No. (%)									52.3
Low	153 (68.0)	647 (43.4)	938 (32.3)	869 (31.5)	775 (27.9)	517 (25.2)	253 (25.0)	86 (17.7)	
Intermediate	22 (9.8)	287 (19.2)	721 (24.9)	727 (26.3)	729 (26.2)	547 (26.6)	289 (28.5)	135 (27.7)	
High	4 (1.8)	156 (10.5)	582 (20.1)	656 (23.7)	737 (26.5)	590 (28.7)	329 (32.5)	195 (40.0)	
Missing	46 (20.4)	401 (26.9)	659 (22.7)	511 (18.5)	538 (19.4)	401 (19.5)	142 (14.0)	71 (14.6)	
Smoking, No. (%)	96 (42.7)	546 (36.6)	1,120 (38.6)	1,153 (41.7)	1,101 (39.6)	816 (39.7)	453 (44.7)	236 (48.5)	9.0
Alcohol use, No. (%)	84 (37.3)	620 (41.6)	1,329 (45.8)	1,477 (53.5)	1,526 (54.9)	1,202 (58.5)	565 (55.8)	240 (49.3)	18.1
House status, No. (%)									32.4
Own house	120 (53.3)	874 (58.6)	1986 (68.5)	1919 (69.5)	2,141 (77.0)	1732 (84.3)	850 (83.9)	384 (78.9)	
Rent house	89 (39.6)	591 (39.6)	856 (29.5)	801 (29.0)	596 (21.4)	304 (14.8)	156 (15.4)	97 (19.9)	
Others	16 (7.1)	26 (1.7)	58 (2.0)	43 (1.6)	42 (1.5)	19 (0.9)	7 (0.7)	6 (1.2)	
Education, No. (%)									
High school or less	118 (52.7)	710 (47.8)	1,235 (42.7)	1,106 (40.1)	1,068 (38.5)	795 (38.7)	343 (33.9)	154 (31.6)	17.7
Missing	1 (0.4)	6 (0.4)	10 (0.3)	4 (0.1)	5 (0.2)	2 (0.1)	1 (0.1)	0 (0.0)	

SMD, standardized mean difference; SD, standard deviation; COPD, chronic obstructive pulmonary disease; BMI, body mass index.

### Crude incidence of COVID-19 infection and admission for COVID-19 infection requiring oxygen

**Figure 2:**
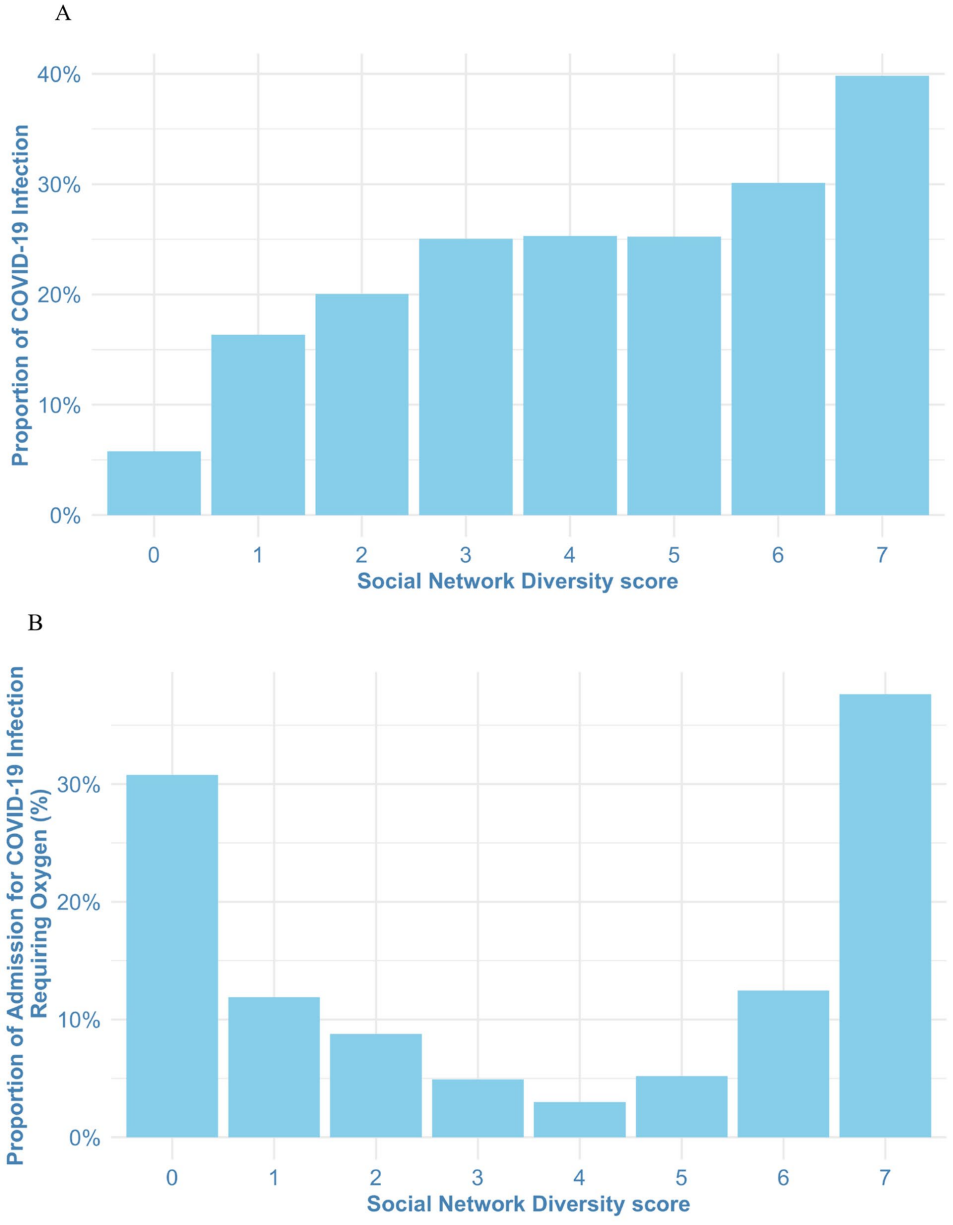
**(A)** Stratified proportions of COVID-19 incidence by social network diversity score. **(B)** Stratified proportions of oxygen-requiring COVID-19 admission by social network diversity score. The incidence of COVID-19 infection exhibited a monotonic increase as the SND score rose **(A)**. The relationship between SND score and the probability of requiring oxygen-requiring admission showed a U-shaped pattern, with the lowest incidence observed at an SND score of 4 **(B)**. COVID-19, Coronavirus disease 2019.

Over the fixed 3-year follow-up period, 3,251 participants (23.7%) developed COVID-19 infection. Supplementary Table 2 shows the baseline characteristics of the 3,251 COVID-19 cases in our study population, stratified by their SND score. Of these, 277 individuals (8.5%) required hospital admission for oxygen supplementation. The incidence of COVID-19 infection exhibited a monotonic increase as the SND score rose (Figure 2). Among participants diagnosed with COVID-19, the relationship between SND score and the probability of requiring oxygen-requiring admission showed a U-shaped pattern, with the lowest incidence observed at an SND score of 4 (Figure 2).

### Association between social network diversity score and the incidence and severity of COVID-19

**Figure 3:**
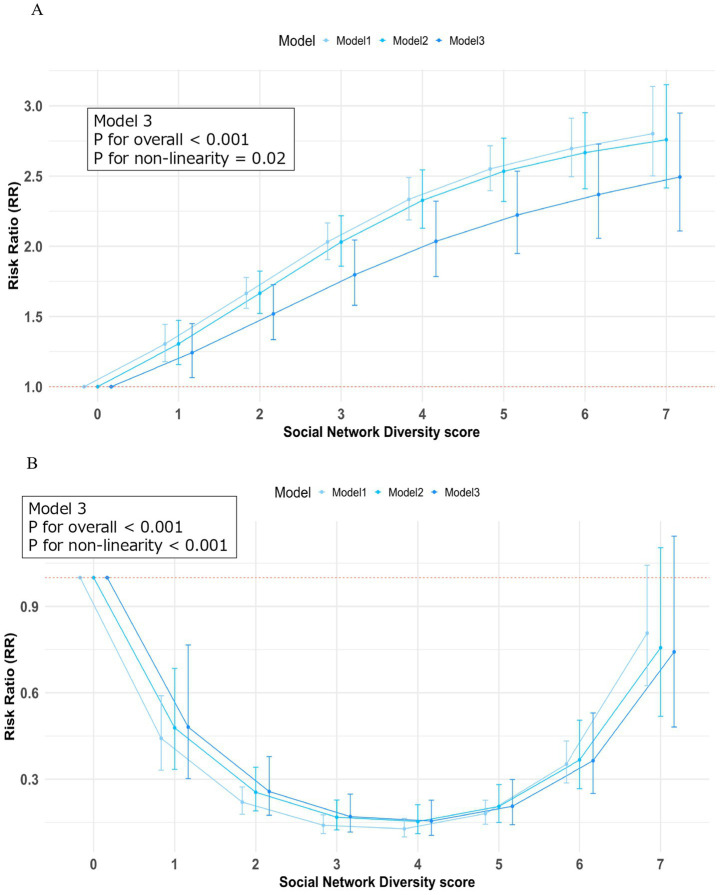
Restricted cubic spline depicting the relationship between social network score and primary outcomes. **(A)** Illustrates the relative risk of COVID-19 infection stratified by SND scores, demonstrating a significantly elevated risk among individuals with an SND score of 7 when compared to the reference population (SND = 0). **(B)** Shows the U-shaped association between SND scores and the probability of developing severe COVID-19 requiring supplemental oxygen therapy among infected participants, with the nadir of risk observed at moderate SND values. COVID-19, Coronavirus disease 2019.

Restricted cubic splines analysis in the fully adjusted model revealed that participants with higher SND scores had an increased risk of COVID-19 infection. Specifically, compared to those with an SND score of 0 (reference), participants with an SND score of 7 exhibited a significantly higher risk of infection (RR 2.49, 95% CI: 2.11–2.95) (Figure 3A). Among COVID-19 patients, restricted cubic splines analysis demonstrated a clear non-linear, U-shaped relationship between SND scores and risk of oxygen-requiring admission among infected participants. Figure 3B graphically illustrates this curve relationship, showing that participants with moderate SND scores (4, 5) had a significantly reduced risk of severe disease (RR 0.15, 95% CI: 0.11–0.30), while those with the highest SND score of 7 showed no significant risk difference (RR 0.74, 95% CI: 0.48–1.14) compared to those with an SND score of 0. To statistically confirm these non-linear relationships, we tested quadratic terms for SND score in the fully adjusted models. For COVID-19 infection, the quadratic term was statistically significant (coefficient: -0.016, SE: 0.005, *p* = 0.003), indicating that while infection risk increased with higher SND scores, the rate of increase decelerated at higher levels. For COVID-19 severity among infected individuals, the quadratic term was highly significant (coefficient: +0.142, SE: 0.015, *p* < 0.001), providing robust statistical evidence for the U-shaped relationship.

Furthermore, even after stratification by age and sex, this trend remained consistent, with higher SND scores associated with increased COVID-19 infection rates (P for interaction 0.50 and 0.75, respectively) (Supplementary Figures 3A,B), while the relationship between SND scores and severity maintained a U-shaped curve (P for interaction 0.11 and 0.16, respectively) (Supplementary Figures 4A,B).

## Discussion

In this large-scale longitudinal cohort study of 13,713 Japanese adults, we found that SND had differential associations with COVID-19 infection and disease severity risk. Higher social network diversity was associated with an increased risk of infection, with a monotonic rise in incidence across increasing levels of network diversity. However, among those who contracted COVID-19, the association between SND and oxygen-requiring admission followed a U-shaped pattern, with the lowest risk observed at moderate levels of network diversity. After adjusting for potential confounders, individuals with the highest SND exhibited a 2.5-fold increased risk of infection compared to those with no social connections, while individuals with moderate social network diversity had an 85% lower risk of oxygen-requiring admission compared to those with no SND. These findings highlight the complex interplay between SND and COVID-19 infection and severity risk, suggesting that while greater SND may elevate infection risk, maintaining moderate SND could confer protective effects against severe COVID-19.

This study identified a significant positive association between SND and COVID-19 infection risk, aligning with existing epidemiological evidence that social contact networks increase transmission risk of respiratory pathogens (19). While diverse social contacts generally function as psychosocial health-promoting factors, they simultaneously increase exposure opportunities to pathogens (2). It should be noted that the association between SND and health outcomes is not strictly linear, with previous reports showing varied associations between SND and upper respiratory tract infection rates. Cohen et al. examined the association between SND and infection risk among 276 healthy volunteers. Their findings revealed that individuals with only 1–3 types of diversity among 12 possible categories demonstrated approximately 4.2 times higher infection risk compared to those with six or more types (5). A previous 12-week prospective study investigating the impact of stress and SND on upper respiratory tract infections suggested that extensive social networks exhibited protective effects against infection under low-stress conditions. However, exposure to multiple different contacts increased infection risk during states of compromised immunity (6). Trade-offs related to early exposure may explain this series of associations. A US birth cohort study showed that early cold infections resulted in lower rates of cold infection later in life, suggesting that immune exposure through contact may play an antagonistic role in subsequent infection development (20).

Considering SARS-CoV-2’s distinctive pathogenicity—characterized by high infectivity and asymptomatic transmission—the infection risks associated with SND may be more pronounced compared to conventional respiratory infections, especially since established immunity may not work effectively against novel infectious diseases (21). The positive association observed in our study between SND and COVID-19 infection risk can be interpreted as reflecting one aspect of these complex interactions. In the context of preventing emerging infectious disease pandemics, while physical isolation is effective for infection prevention due to the lack of prior immune acquisition opportunities, the protective role of partial immunity may be supplemented by increased diversity of social contacts and could potentially work preventively from a long-term perspective (22).

Regarding disease severity, our findings revealed a U-shaped relationship between SND and COVID-19 severity, with moderate SND consistently associated with reduced risk of severe COVID-19 across all models. Previous research has linked limited SND to increased health risks, including cardiovascular and respiratory diseases (8). Indeed, mortality risk associated with low SND has been reported to be comparable to that of smoking (23). Studies have shown that individuals with higher SND exhibit lower mortality risk, while those with contact frequency less than once monthly show approximately 20% increased risk of premature death (24). The Chicago Health and Aging Project, focusing on adults aged 65 and older, demonstrated that higher SND independently correlated with lower mortality rates and better cognitive and physical function (25). In the context of infectious diseases, SND may suppress inflammation and enhance antiviral responses (26).

The biological foundation for this association lies in physiological pathways between social connections and clinical health, where high social support correlates with improved functional immunity, such as enhanced natural killer cell cytotoxicity, while isolation correlates with decreased immune function (27). SND serves as an indicator of social integration and engagement, with increased participation opportunities correlating with reduced mortality, functional disability, and mental health risks, demonstrating long-term benefits beyond what network size alone can capture. Our findings indicated that moderate levels of SND were associated with decreased severity risk. The balance between immune system activation might explain this nonlinear relationship through social contact and increased exposure opportunities. These insights suggest the need to consider an appropriate balance between the benefits and risks of SND when developing public health policies during pandemics. Importantly, these differential associations (monotonic for infection, U-shaped for severity) remained robust even after extensive adjustment for key demographic factors, including age and sex. Furthermore, our stratified analyses showed these findings were consistent across different age and sex groups, highlighting the generalizability of this complex relationship.

Our findings suggest the need for more nuanced public health policies that move beyond one-size-fits-all social distancing mandates and instead consider the differential risks associated with SND. This suggests a targeted, two-pronged strategy. First, individuals with very high SND (e.g., essential workers, those with extensive community roles) face the greatest infection risk and require targeted interventions. Rather than advising isolation, which could eliminate the social connections that may protect them from severe disease, interventions should focus on making their necessary contacts safer through measures such as prioritizing vaccination, promoting high-quality masking, improving ventilation in workplaces, and utilizing rapid testing (28). Second, for individuals with very low SND (e.g., isolated older adults), who are at the highest risk for severe outcomes, public health strategies should aim to safely increase their social connections to a moderate level (29). Furthermore, digital technology is a crucial tool. Promoting the use of video calls, online community groups, and telehealth platforms can facilitate these “hybrid social interactions.” This approach can provide the documented psychosocial and immunological benefits of social connection while effectively minimizing the risk of physical exposure to pathogens (30, 31). These balanced strategies, tailored to an individual’s level of social integration, will be critical for building resilience in future pandemics.

### Limitations

This study has several important limitations. First, our primary outcome measures and SND scores were based on self-reported surveys rather than clinical or administrative records. Specifically, the self-reported items used to assess COVID-19 infection and oxygen-requiring hospitalization were not validated against medical records or registry data in this study. This reliance on self-reported data may have introduced recall bias or misclassification, as participants might have inaccurately remembered their past infection history, hospitalization details, or frequency of social contacts. Second, while our analyses adjusted for various demographic and clinical variables, the observational nature of the study makes it impossible to rule out unmeasured or inadequately measured confounding factors. Crucially, our dataset lacked precise information on the exact timing of infection relative to the varying waves of the pandemic, as well as detailed vaccination status (e.g., number of doses and timing). The absence of these data means we could not fully account for their potential confounding effects on the observed associations between social contact and COVID-19 outcomes. Third, the SND score used in this study was originally developed for this analysis and has not been extensively validated in other populations or settings. Since social interaction patterns can vary significantly across different cultural and demographic contexts, the generalizability of our findings to populations outside of Japan remains uncertain. Although we designed the score to closely align with established methodologies in social network research (5) to ensure construct validity, future studies are needed to validate this specific scoring system in diverse cohorts. Fourth, we must acknowledge the potential for selection bias due to attrition, as approximately 51% of the original cohort was lost to follow-up. Although we adjusted for these sociodemographic factors in our multivariable models to minimize bias, the final cohort may be more socially stable than the general population. Therefore, it is possible that those who remained in the study differed in terms of health consciousness, social network characteristics, or socioeconomic status compared to those who dropped out. Fifth, our analysis relied on a single baseline measurement of SND in 2020. We did not account for potential changes in social behavior over the three-year follow-up period, which may have fluctuated due to evolving public health policies, vaccination status, or changes in individual risk perception. This limitation may have introduced non-differential exposure misclassification, potentially biasing the effect estimates toward the null.

## Conclusion

In this large-scale longitudinal cohort study of 13,713 Japanese adults, we demonstrated that social network diversity had differential associations with COVID-19 infection risk and disease severity. Higher social network diversity was associated with increased infection risk, whereas the association with disease severity followed a U-shaped pattern, indicating that moderate social network diversity was protective against oxygen-requiring admission. The current study suggests that maintaining moderate social connections may be protective against severe COVID-19 outcomes, highlighting the importance of balanced approaches to social distancing measures during infectious disease outbreaks. These findings underscore the potential benefit of strategies that preserve moderate social connectivity while implementing appropriate preventive measures during future pandemics.

## Data Availability

The data analyzed in this study is subject to the following licenses/restrictions: the data used in this study are not available in a public repository because they contain personally identifiable or potentially sensitive patient information. Based on the regulations for ethical guidelines in Japan, the Research Ethics Committee of the Osaka International Cancer Institute has imposed restrictions on the dissemination of the data collected in this study. Requests to access these datasets should be directed to all data enquiries should be addressed to the person responsible for data management, TT at the following e-mail address: tabuchitak@gmail.com.
