# Supplementary material for: Social network diversity and COVID-19 infection and severity risk: a longitudinal population study
Source: Front Public Health. 2026 Jan 13;13:1730268. doi: 10.3389/fpubh.2025.1730268 (PMC12834764; doi:10.3389/fpubh.2025.1730268)

**Supplemental Material**

**eTable 1.** Comparative analysis of eligible and excluded cases in the study

**eTable 2.** Baseline characteristics of participants with incidence of COVID-19, stratified by Social Network Diversity Score

**eFigure 1.** Directed acyclic graph (DAG) depicting potential causal pathways between social network diversity score and health outcomes with associated confounders

**eFigure 2**. Distribution of Social Network Diversity score in this study population

**eFigure 3-A**. Association between Social Network Diversity score and the incidence of COVID-19, stratified by age

**eFigure 3-B**. Association between Social Network Diversity score and the incidence of COVID-19, stratified by sex

**eFigure 4-A**. Association between Social Network Diversity score and the severity of COVID-19, stratified by age

**eFigure 4-B**. Association between Social Network Diversity score and the severity of COVID-19, stratified by sex

**eTable 1.** Comparative analysis of eligible and excluded cases in the study

|  | Eligible participants | Excluded participants | SMD (Percentage Points) |
| --- | --- | --- | --- |
| Number of the participants | 13,713 | 14,287 |  |
| Age (years), mean (SD) | 53.2 (15.7) | 44.4 (17.7) | 52.5 |
| Female gender, No. (%) | 6,357 (46.4) | 7,687 (53.8) | 14.9 |
| Body Mass Index, mean (SD) kg/m^2^ | 22.6 (7.7) | 22.5 (7.4) | 0.8 |
| Previous medical history |  |  |  |
| Hypertension, No. (%) | 3,796 (27.7) | 2,884 (20.2) | 17.6 |
| Diabetes, No. (%) | 1,217 (8.9) | 1,130 (7.9) | 3.5 |
| COPD, No. (%) | 244 (1.8) | 398 (2.8) | 6.7 |
| Asthma, No. (%) | 1,587 (11.6) | 2,113 (14.8) | 9.5 |
| Income, No. (%) |  |  | 5.3 |
| Low | 4,238 (30.9) | 4,149 (29.0) |  |
| Intermediate | 3,457 (25.2) | 3,737 (26.2) |  |
| High | 3,249 (23.7) | 3,297 (23.1) |  |
| Missing | 2,769 (20.2) | 3,104 (21.7) |  |
| Smoking, No. (%) | 5,521 (40.3) | 4,777 (33.4) | 14.2 |
| Alcohol use, No. (%) | 7,043 (51.4) | 6,403 (44.8) | 13.1 |
| House status, No. (%) |  |  | 16.8 |
| Own house | 10,006 (73.0) | 9,323 (65.3) |  |
| Renthouse | 3,490 (25.5) | 4,692 (32.8) |  |
| Others | 217 (1.6) | 272 (1.9) |  |
| Education, No. (%) |  |  |  |
| High school or less | 5,529 (40.3) | 6,059 (42.4) | 5.1 |
| Missing | 29 (0.2) | 50 (0.3) |  |

SMD, standardized mean difference; SD, standard deviation; COPD, chronic obstructive pulmonary disease; BMI, body mass index

**eTable 2.** Baseline characteristics of participants with incidence of COVID-19, stratified by Social Network Diversity Score

|  | 0 | 1 | 2 | 3 | 4 | 5 | 6 | 7 | SMD  (Percentage Points) |
| --- | --- | --- | --- | --- | --- | --- | --- | --- | --- |
| Number of the participants | 13 | 244 | 581 | 692 | 703 | 519 | 305 | 194 |  |
| Age (years), mean (SD) | 49.7 (17.2) | 48.4 (14.6) | 46.0 (14.6) | 46.7 (15.2) | 47.3 (15.1) | 51.1 (15.1) | 48.2 (16.7) | 40.8 (14.4) | 22.6 |
| Female gender, No. (%) | 5 (38.5) | 118 (48.4) | 226 (38.9) | 356 (51.4) | 387 (55.0) | 258 (49.7) | 114 (37.4) | 53 ( 27.3) | 22.7 |
| Body Mass Index, mean (SD) kg/m^2^ | 20.2 (4.5) | 22.5 (4.1) | 22.7 (5.0) | 22.3 (3.7) | 22.4 (5.6) | 22.4 (3.5) | 23.1 (4.4) | 22.5 (3.5) | 16.8 |
| Previous medical history |  |  |  |  |  |  |  |  |  |
| Hypertension, No. (%) | 5 (38.5) | 65 (26.6) | 131 (22.5) | 161 (23.3) | 145 (20.6) | 127 (24.5) | 77 (25.2) | 66 ( 34.0) | 15.4 |
| Diabetes, No. (%) | 4 (30.8) | 23 ( 9.4) | 44 ( 7.6) | 44 ( 6.4) | 44 ( 6.3) | 36 ( 6.9) | 38 (12.5) | 40 ( 20.6) | 26.6 |
| COPD, No. (%) | 2 (15.4) | 11 ( 4.5) | 7 ( 1.2) | 11 ( 1.6) | 4 ( 0.6) | 5 ( 1.0) | 16 ( 5.2) | 21 ( 10.8) | 26.0 |
| Asthma, No. (%) | 3 (23.1) | 37 (15.2) | 69 (11.9) | 90 (13.0) | 100 (14.2) | 71 (13.7) | 52 (17.0) | 47 ( 24.2) | 14.0 |
| Income, No. (%) |  |  |  |  |  |  |  |  | 71.6 |
| Low | 11 (84.6) | 105 (43.0) | 160 (27.5) | 187 (27.0) | 158 (22.5) | 97 (18.7) | 68 (22.3) | 36 ( 18.6) |  |
| Intermediate | 0 ( 0.0) | 61 (25.0) | 147 (25.3) | 189 (27.3) | 198 (28.2) | 139 (26.8) | 85 (27.9) | 64 ( 33.0) |  |
| High | 0 ( 0.0) | 29 (11.9) | 144 (24.8) | 194 (28.0) | 229 (32.6) | 200 (38.5) | 109 (35.7) | 75 ( 38.7) |  |
| Missing | 2 (15.4) | 49 (20.1) | 130 (22.4) | 122 (17.6) | 118 (16.8) | 83 (16.0) | 43 (14.1) | 19 ( 9.8) |  |
| Smoking, No. (%) | 4 (30.8) | 82 (33.6) | 199 (34.3) | 257 (37.1) | 251 (35.7) | 193 (37.2) | 129 (42.3) | 100 (51.5) | 14.7 |
| Alcohol use, No. (%) | 4 (30.8) | 108 (44.3) | 262 (45.1) | 362 (52.3) | 384 (54.6) | 293 (56.5) | 147 (48.2) | 84 (43.3) | 19.3 |
| House status, No. (%) |  |  |  |  |  |  |  |  | 41.3 |
| Own house | 5 (38.5) | 133 (54.5) | 375 (64.5) | 427 (61.7) | 482 (68.6) | 401 (77.3) | 231 (75.7) | 145 (74.7) |  |
| Renthouse | 4 (30.8) | 110 (45.1) | 196 (33.7) | 255 (36.8) | 210 (29.9) | 111 (21.4) | 71 (23.3) | 46 (23.7) |  |
| Others | 4 (30.8) | 1 (0.4) | 10 (1.7) | 10 (1.4) | 11 (1.6) | 7 (1.3) | 3 (1.0) | 3 (1.5) |  |
| Education, No. (%) |  |  |  |  |  |  |  |  |  |
| High school or less | 7 (53.8) | 110 (45.1) | 244 (42.0) | 259 (37.4) | 230 (32.7) | 189 (36.4) | 93 (30.5) | 68 ( 35.1) | 19.7 |
| Missing | 0 ( 0.0) | 1 ( 0.4) | 3 ( 0.5) | 2 ( 0.3) | 2 ( 0.3) | 1 ( 0.2) | 0 ( 0.0) | 0 ( 0.0) |  |

SMD, standardized mean difference; SD, standard deviation; COPD, chronic obstructive pulmonary disease; BMI, body mass index

**eFigure 1**. Directed acyclic graph (DAG) depicting potential causal pathways between social network diversity score and health outcomes with associated confounders


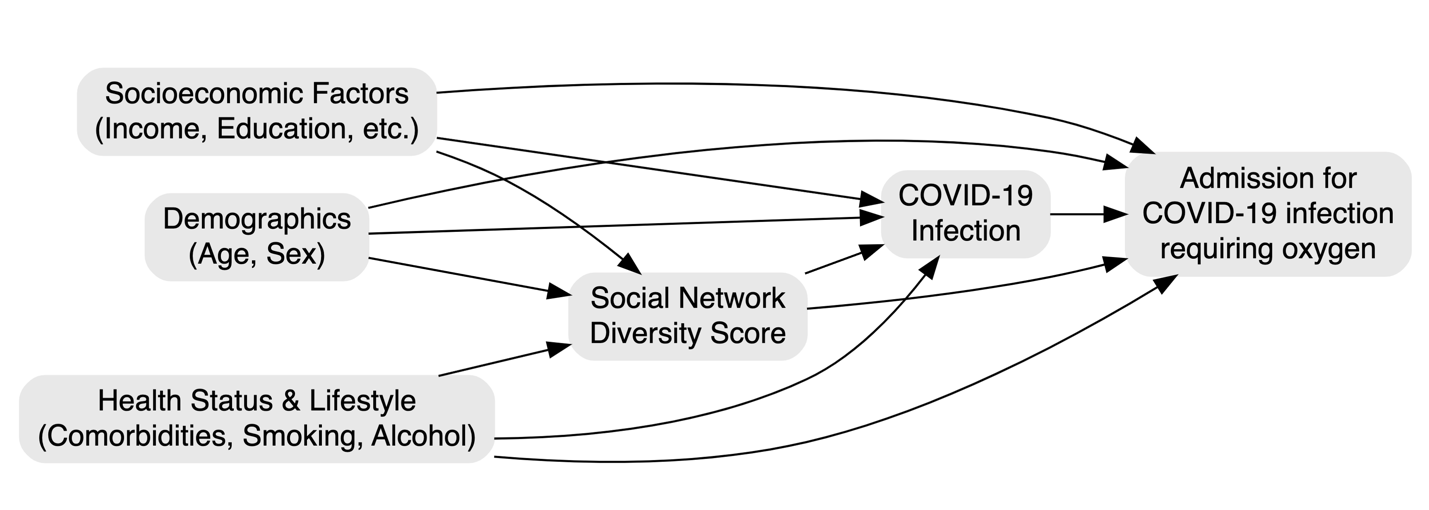


**eFigure 2**. Distribution of Social Network Diversity score in this study population


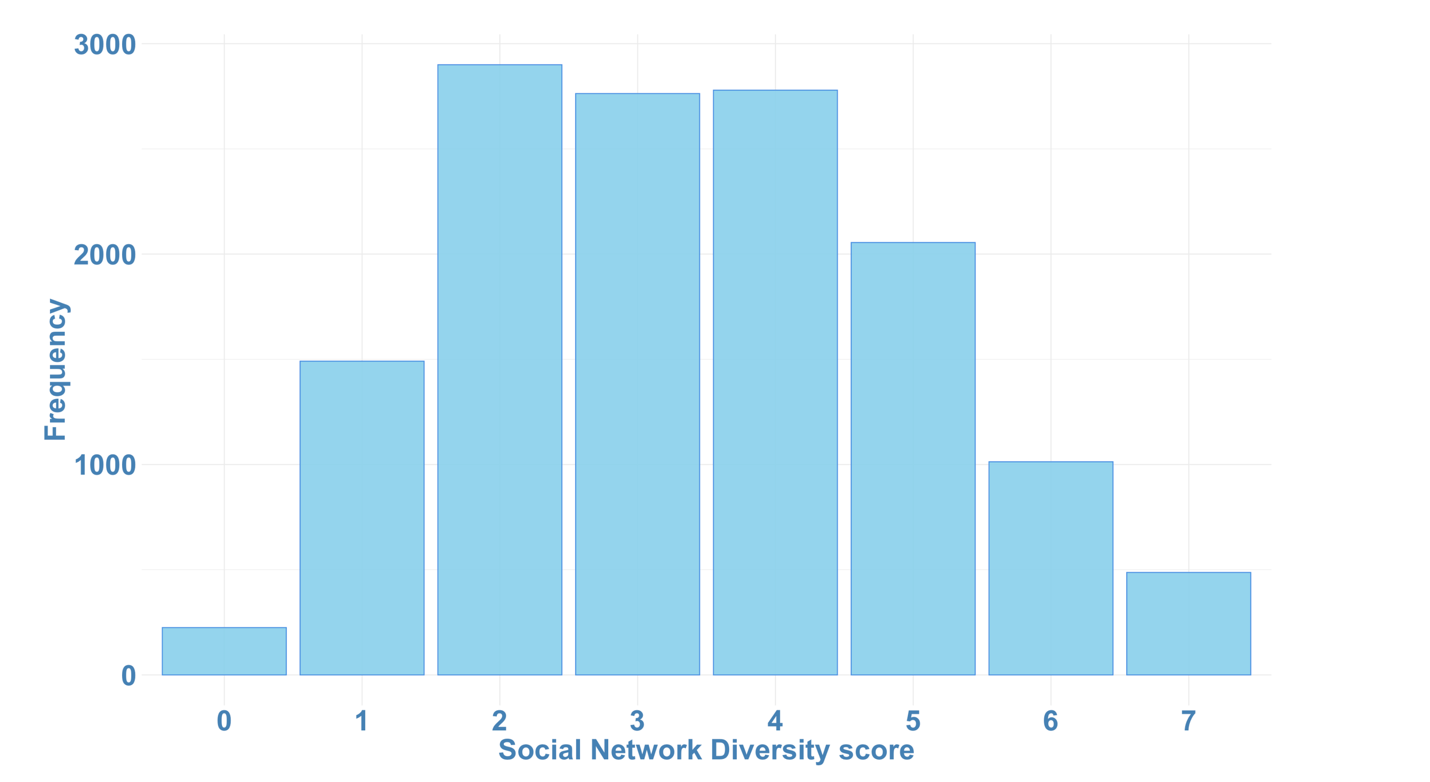


**eFigure 3-A**. Association between Social Network Diversity score and the incidence of COVID-19, stratified by age


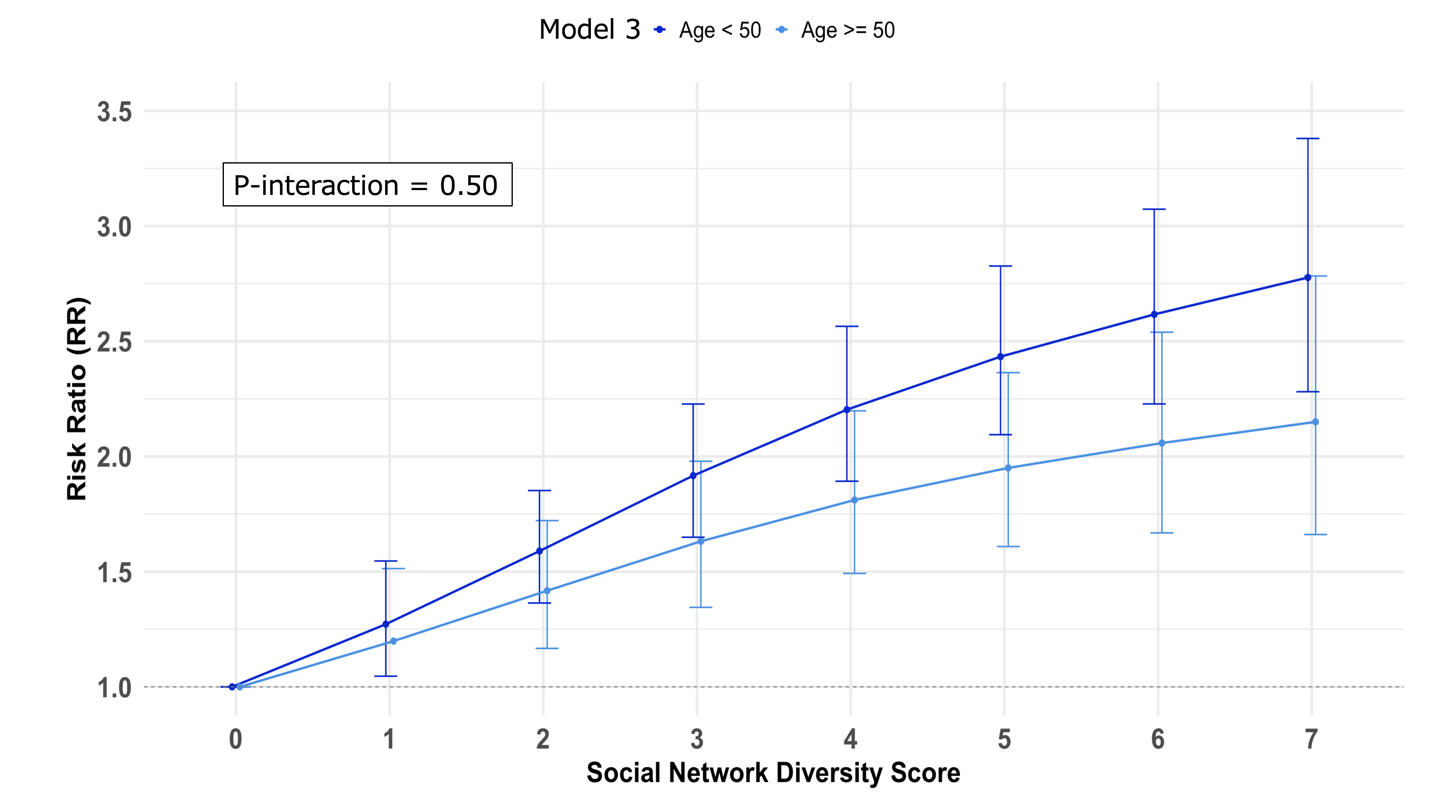


**eFigure 3-B**. Association between Social Network Diversity score and the incidence of COVID-19, stratified by sex


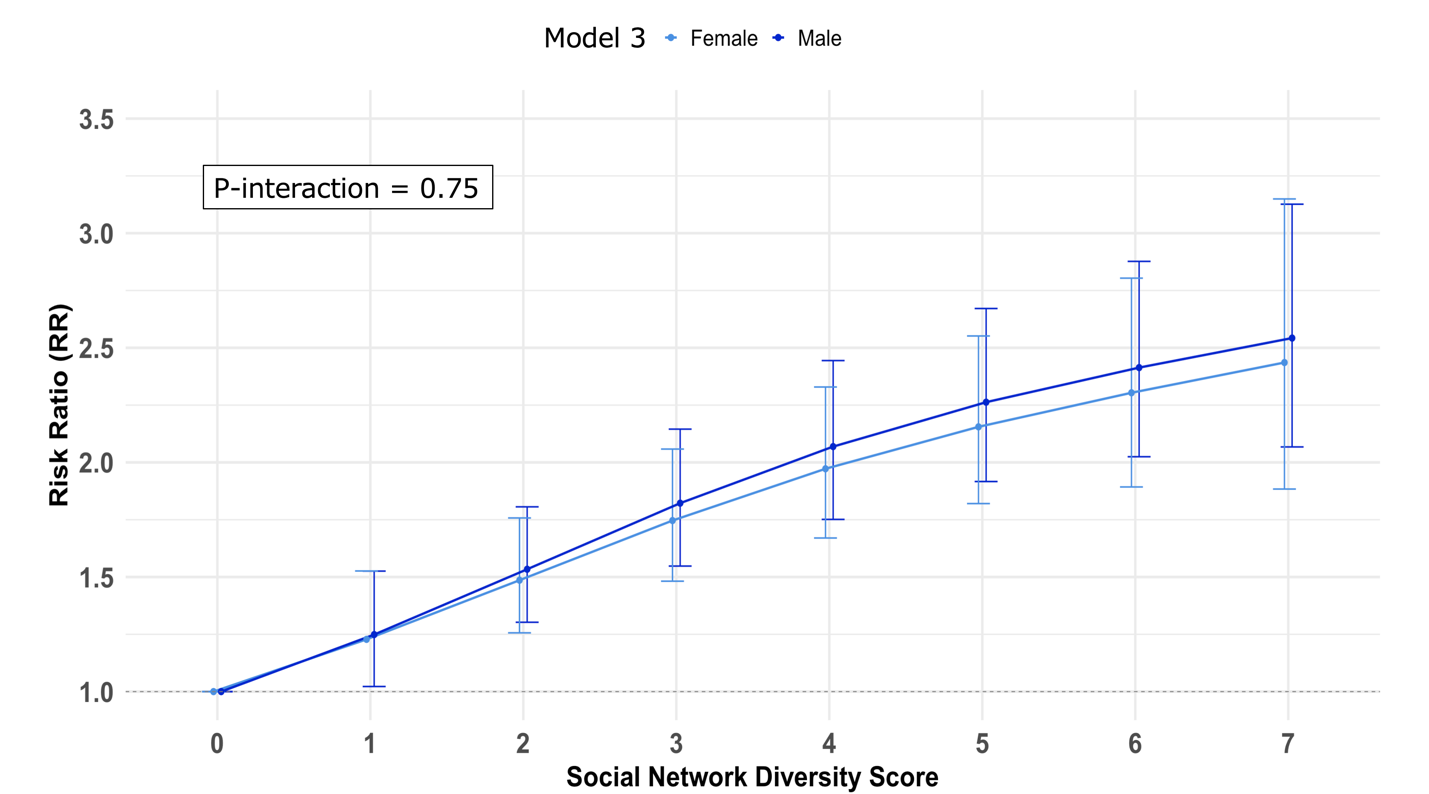


**eFigure 4-A**. Association between Social Network Diversity score and the severity of COVID-19, stratified by age


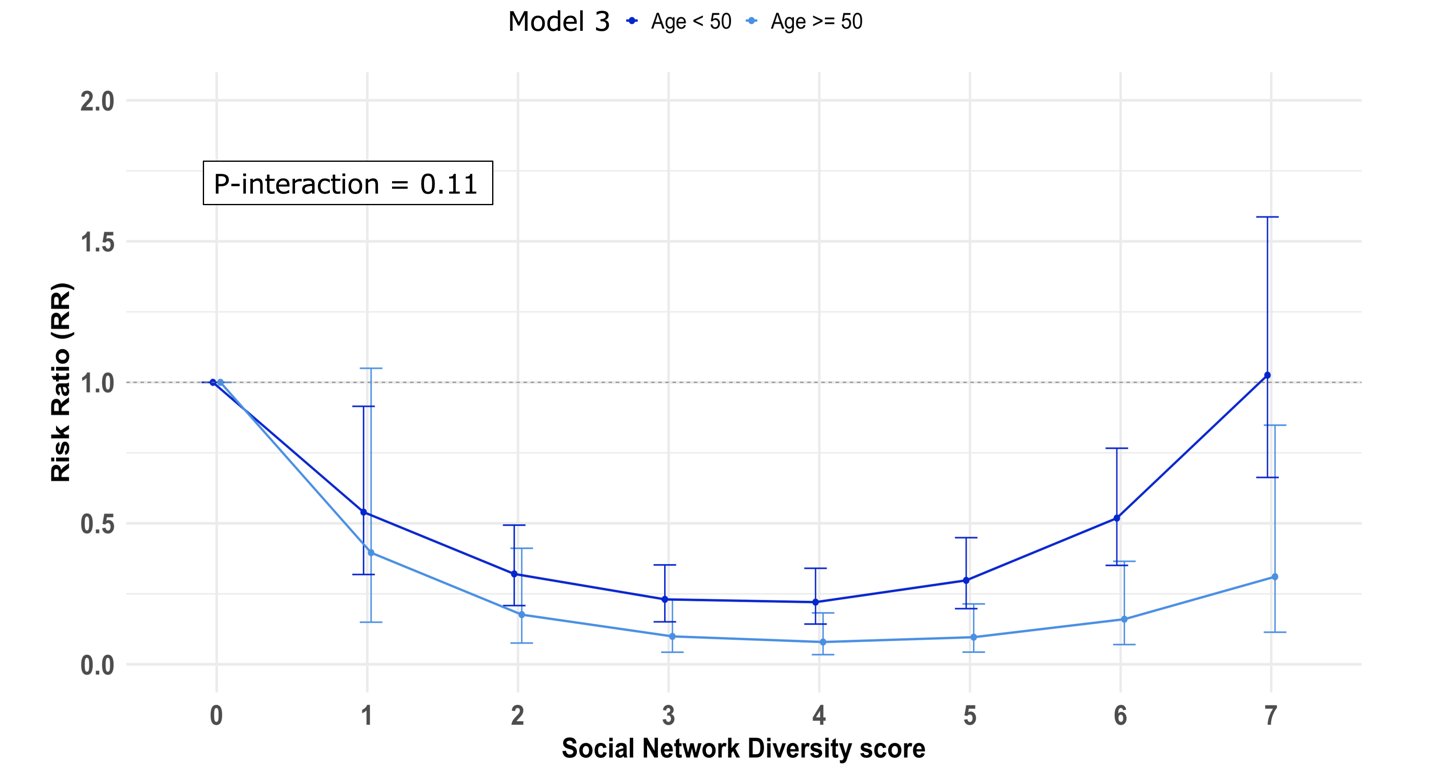


**eFigure 4-B**. Association between Social Network Diversity score and the severity of COVID-19, stratified by sex


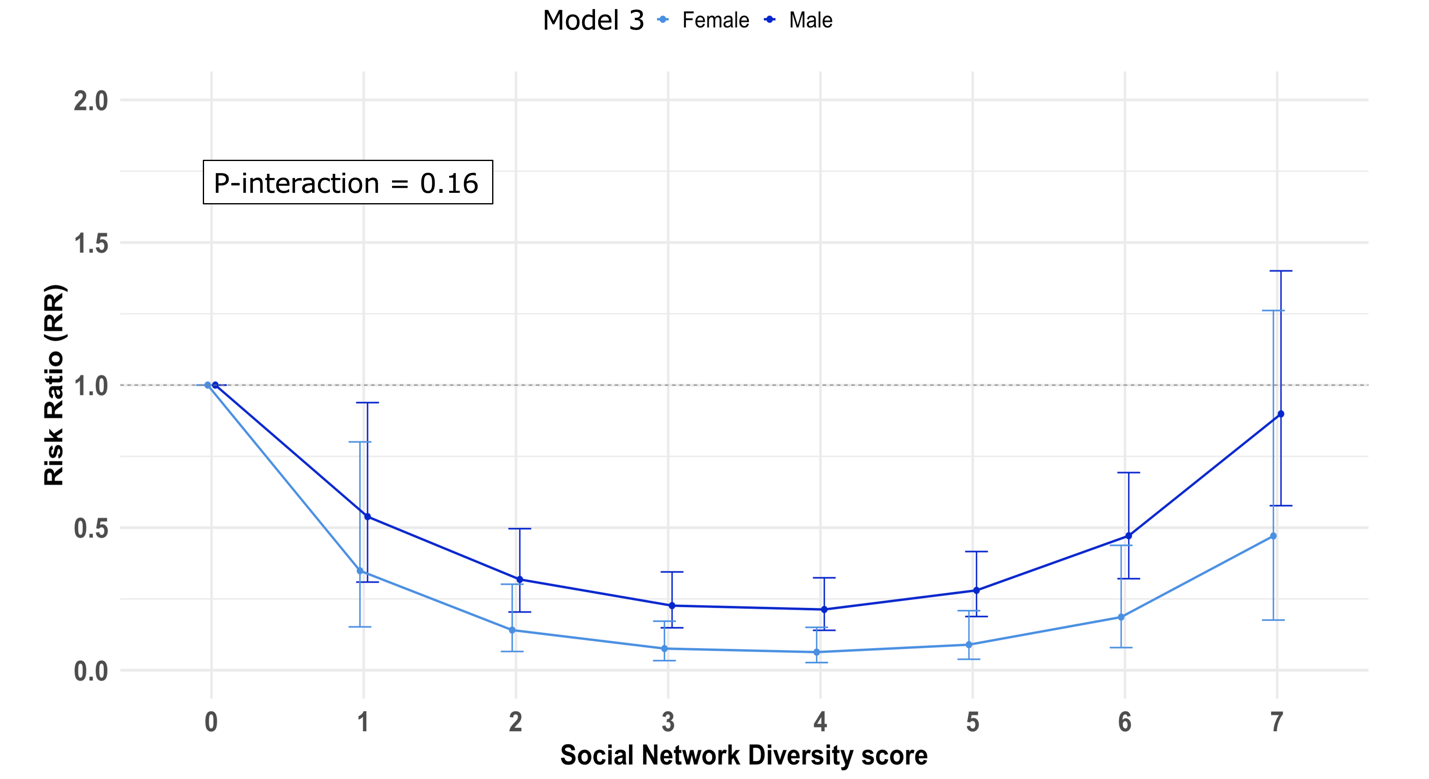

Supplement: Supplementary file 1 [file Supplementary_file_1.docx]
